# Labeling in a California Latinx community: public health implications for youth and role in community narratives

**DOI:** 10.1186/s12889-025-23598-z

**Published:** 2025-07-23

**Authors:** Elodia Caballero, Alexandra Minnis, Deepika D. Parmar, Melissa Zerofsky, Megan Comfort, Marissa Raymond-Flesch

**Affiliations:** 1 Department of Obstetrics and Gynecology, Stanford University School of Medicine, 453 Quarry Road, Palo Alto, CA 94304 USA; 2 RTI International, Berkeley, CA USA; 3 Division of Adolescent and Young Adult Medicine, Department of Pediatrics, Lee Institute for Health Policy Studies, University of California, San Francisco, CA USA; 4 Department of Pediatrics, University of California, San Francisco, CA USA

**Keywords:** Labeling, Social connection, Adolescent, Latinx, Stigma

## Abstract

**Background:**

Prior research demonstrates that Latinx youth disproportionately receive negative labels from parents, peers, and teachers. Negative labels are stigmatizing and often result in rejection or devaluation of the labeled person which can be pivotal within the developmental window of adolescence. Prior research has already shown that experiences of exclusion and social rejection can have detrimental impacts on mental and physical health. To this end, it is paramount to understand how labeling is wielded, especially among those most vulnerable, to devise thoughtful solutions. This study examines how labeling is operationalized within a Latinx community and consequences of such labels on health-protective behaviors, wellbeing, peer networks and school engagement.

**Methods:**

In-depth interviews were conducted with 39 adolescents and 20 mothers from a predominantly Latinx and immigrant agricultural community in California. Teams of coders completed iterative rounds of memoing and team meetings to identify themes and develop a code book of inductive and deductive codes. The team completed iterative rounds of coding to refine the codebook and review code discrepancies to ensure fidelity of coding. After coding of mother and adolescent transcripts an additional round of memos and team meetings focused on similarities and differences in youth and mothers’ perspectives about labeling.

**Results:**

Dichotomous labeling of “good” and “bad” arose spontaneously in all mothers and most youth interviews and were pervasive within schools and the broader community. Youth labeled as “bad” experienced limited educational opportunities, exclusion from peers, and community disengagement. While “good kid” labels resulted in educational opportunity and community acceptance these labels also limited health protective-behaviors in some youth, including foregoing contraception. Participants pushed back on negative labeling when it was applied to close family or community acquaintances.

**Conclusions:**

Targeted interventions that foster social belonging and connection rather than exclusion may facilitate health protective behaviors and have positive implications for future trajectories among youth.

**Supplementary Information:**

The online version contains supplementary material available at 10.1186/s12889-025-23598-z.

## Background

Adolescence is a highly dynamic period characterized by rapid physical, cognitive and social developmental changes [1]. Whereas structural determinants of health such as wealth, income, education, health-care access, and employment opportunities, have salient health impacts across an individual’s lifetime, proximal and intermediate determinants, such as social connectedness to peers, families and school are also crucial in shaping health-related behaviors and educational trajectories among youth [2, 3]. Latinx youth and youth in immigrant families face unique challenges in achieving social connectedness under the load of acculturative stress which cumulatively contributes to health disparities [4–6].

Social connections are one of several important factors that protect against poor health outcomes in adolescence. Safe and supportive families, schools, and community as well positive and supportive peers are crucial to helping young people develop to their full potential and attain the best health in adulthood [7]. Likewise, disconnection and exclusion from supportive networks can have detrimental effects [8]. As demonstrated by Nurd-Sharps and Lewis (2018), youth disconnected from social support and networks are twice as likely to live in poverty [9]. These youth are also at higher risk for undesired pregnancy, incarceration, high school disenrollment and mental health conditions [9–11].

Labeling theory postulates that self-identity can be influenced labels, or the terms used to describe or classify people [12]. It argues once individuals are labeled as deviants, their navigation of future scenarios are influenced by these labels [12, 13]. This is similar to other literature highlighting the impacts of social rejection, which may be particularly acute in the critical developmental window of adolescence [14]. Initial studies of labeling focused on formal labeling, defined as a labeling dispensed by a person or organization with official status such as the label of felon imposed by the justice system [15]. However, recent literature underscores the relative importance of informal and perceived labeling [16]. Informal labeling can result from stigma associated with formal labels, or it can arise through social networks in the absence of formal labels [15]. For adolescents, informal negative labeling by the community, family, and peers can be equally or even more impactful on increased recidivism when compared with formal labeling through the justice system [17].

Research, largely focused within the school setting, has shown Latinx youth are more likely to receive negative social labels for age normative behaviors than non-Latinx white youth, resulting in racial and ethnic disparities in discipline [18]. To this end, school discipline practices can become almost exclusively a form of labeling and social control that disproportionately targets and imposes negative labels on Latinx youth. Additionally, holding multiple marginalized identities or experiencing several acts of social rejection intersects and compounds negative impacts on mental and physical health [14, 19, 20].

A downfall of labeling is its oversimplification of the human condition, often enforcing rigid identity binaries and confining narratives. While the literature supports that Latinx youth are overwhelming recipients of exclusionary negative labels, very few studies examine how labeling is operationalized among Latinx youth and their families. Literature on the subsequent impact on youth’s health and wellness is also scarce. This is critical as the disparate impact and outcomes of exclusionary labels may widen existing health disparities for minoritized youth who already experience higher rates of adverse childhood experiences (ACEs) and other traumas [21, 22].

Understanding how youth perceive and react to harmful labels can help to uncover strategies to mitigate the use of labels in different settings, thereby diminishing their impact on psychological wellbeing and connection to preventive health services.

The present analysis is part of *A Crecer*: The Salinas Teen Health Study. The overarching objectives of *A Crecer*, a longitudinal mixed methods cohort study, are to examine the multi-level factors that influence Latinx adolescents’ wellbeing, as well as the onset of youth violence, mental health, and sexual health behaviors associated with teen childbearing within the primarily Latinx agricultural community of Salinas California. *A Crecer* is informed by the socioecological model which postulates that human development occurs through reciprocal interactions between individuals or groups in the immediate environment (families, peers, individual interactions), which are shaped by the larger environmental context (schools, policies, etc.). The qualitative interviews that are integrated into *A Crecer* are designed to provide additional context about the environmental factors influencing youth health and wellbeing [23]. The objective of the present analysis is to investigate how community and peer labeling affect adolescents’ relationships with peers, engagement in school, health behaviors, and support from adultsfrom the perspectives of adolescents and mothers in Salinas, CA.”

## Methods

### Setting

Salinas, CA is the urban center of the Salinas Valley, the agricultural region on California’s central coast. The agricultural industry is the largest employer in the region. Salinas is 80% Latinx, 11% Non-Latinx white and 38% first generation immigrants [24]. 17% of the population lives below the federal poverty level and the city’s public schools have universal free breakfast and lunch programs due to rates of childhood poverty [25]. Civic engagement and community involvement has been shown to be of high importance among Salinas residents through attendance at public hearings, advisory boards, and community planning meetings to support programs and policies aim to reduce poverty rate [25].

### Study population and design

#### Youth study participants: A Crecer (To Grow)

The Salinas Teen Health Study, is a community-engaged, mixed methods, longitudinal cohort study of 599 primarily Latinx adolescents during their transition from eighth grade into high school and beyond. The overarching objectives of this mixed methods study are to examine the multi-level factors that influence Latinx adolescents’ wellbeing, as well as the onset of youth violence, mental health, and sexual health risks associated with teen childbearing. The objective of this analysis is to investigate how community and peer labeling affect relationships with peers, engagement in school, health behaviors, and support from adults in the *A Crecer* cohort. *A Crecer* was initiated by community members and Department of Health Officials who were interested in strength-based framing and approaches to the community’s challenges. To operationalize a strength-based approach the research team sought to investigate not only risk factors for poor health outcomes, but also individual, family, community, and cultural factors that support youth health and wellbeing. Our research team sought to maintain this strengths-based lens though iterative engagement with our community advisors, youth advisors, and community co-investigators wherein we solicited input on our research approach, research questions, and interpretation of our results.

Participants were eligible if they were in 8th grade, between 12 and 15 years old, spoke English or Spanish, and planned to live in Salinas for at least one year following enrollment. From November 2015 to March 2017 bilingual and bicultural Salina-based research assistants enrolled youth in-person from the four middle schools in the Salinas Union High School District. This sample represented approximately one third of all 8th graders in the district. Researchers attended multiple meetings with school district administrators, during which they presented the project and how the study goals aligned with those of the school district for supporting student success. 800 participants had parent obtained permission to enroll. Of those, 200 declined to participant and 1 youth determined to be ineligible with the final study sample being 599. The main reason for declining participation was disinterest. Further information on our focus groups and community engagement strategies that informed our recruitment approach and research plans have been published previously [26].

All 599 participants completed interviewer-administered questionnaires with additional data on sensitive topics collected via audio computer-assisted self-interviewing. Interview guides were developed from an iterative process involving informational interviews with community stakeholders.

We invited a subsample of 39 participants (20 girls and 19 boys) to participate in two in-depth interviews (78 interviews total, approximately 60 min or less) occurring from 2016 to 2019 at approximately months six and eighteen of their study participation. Within each school we randomly selected in-depth interview participants within subgroups defined by self-identified gender, socioeconomic and educational measures. Interview topics included adolescents’ future aspirations, support systems, and relationships. Participants received a $20 payment for each study visit.

#### Mother study participants

The Salinas Teen Health research team completed an additional 20 interviews (approximately 90 min or less) with mothers of youth in the study cohort in 2019. Mothers were purposely sampled to include US born and immigrant participants as well as mothers with a variety of educational backgrounds. Sampling was also based on the self-identified gender of the child as well as their immigrant generation status. All interviews with mothers were conducted by bilingual bicultural research assistants in Spanish in accordance with the mothers’ preferences. These interviews focused on mothers’ experiences parenting in Salinas, their goals for the health and wellbeing of their children, and the formal and informal supports that they and their children have within the community.

Progress towards data saturation was reviewed after every 10 interviews for both mother and adolescent interviews. Saturation was reached at 40 interviews for youth and 25 for mothers. Transcripts were not returned to participants. Instead, interviewers did a quality check on them for accuracy after transcription and translation. The study maintains both a community advisory board, parents, and community-based organizations, as well as a youth advisory board. These advisory boards reviewed the findings and provided feedback.

The RTI and UCSF Institutional Review Boards approved all study activities. Parent permission and minor assent was obtained for all adolescent participants. Mothers provided written informed consent.

### Qualitative analyses

#### Youth data

Qualitative analyses were conducted by six team members. The coding team conducted initial rounds of thematic analyses which they further developed in thematic memos. They discussed and distilled these themes into inductive and deductive codes through a series of team meetings. The team’s iterative approach was modeled after Bingham and colleagues. Initial deductive codes drawn from the socioecological model which informs the overarching mixed methods study [27, 28]. An inductive approach to the analysis was consistent with the study’s overall community-engaged methods which treat our participants as experts in their community and lived experiences and holds participants as a co-producer of knowledge. The phenomenon of dichotomous labeling and its impact on youth arose de novo in both maternal and youth interviews (i.e. questions about labeling were not built into the interview guides). In our coding scheme the labeling code could be applied to any community member including mother or youth participants and other children and adults in the community. After drafting the code book, all team members then applied the code book to the same two transcripts allowing further refinement of the code book and assessment of inter-coder agreement. One or two team members then coded each of the remaining transcripts.

#### Mothers’ data

Qualitative analyses were conducted by a team of 4 researchers with two additional senior investigators (AM and MC) reviewing the codebook and offering insights to common themes between youth and maternal data. Similar processes were followed with initial rounds of thematic analysis resulting in thematic memos which the team discussed and distilled into inductive and deductive codes at team meetings. Two transcripts were then coded by all team members resulting in further refinement of the code book and assessment of inter-coder agreement using Dedoose software. The remaining transcripts were coded by two team members each. Labeling and its impact on youth was identified as a phenomenon parallel to the labeling theme identified in the youth data during early thematic analysis. The code “Labeling” was defined in parallel to the code used in the youth data. The study teams worked together to examine the fidelity of the code across both youth and mother data as multiple viewpoints triangulate and further validate the salience of this theme in the community. Subsequent memos focused on similarities and differences in youth and mothers’ perspectives about labeling.

## Results

The characteristics of the study sample which include youth (*n* = 39), and mothers (*n* = 20) are shown in Table 1. Youth in the study were primarily second generation (64%) with 80% having lived in the US their entire life. Approximately, 30% were children of an agriculture worker, with 48% having a mother with less than a high school education. All mothers in the sample were first-generation primarily Spanish speaking; 80% have less than a high school education. The average age of mothers at immigration was 19.9 years (range: 13–29 years). The average number of children per mother was 3 (range: 1–5). Demographic data for parents (years since immigration, number of children, employment) and youth (gender, US born or not, mother’s employment, middle school attended) were examined for trends related to the reported results without clear patterns arising.

**Table 1:** Characteristics of youth and mother participants

	**Children (** ***N*** **=39)**		**Mothers (** ***N*** **=20)**		
	***N***	**%**	***N***	**%**	**Mean (range)**
*Self-identified gender*					
Boy	19	48.7			
Girl or Woman	20	51.3	20	100	
*Immigrant generation*					
1st gen	8	20.5	20	100	
2nd gen	25	64.1			
3rd gen +	5	12.8			
Unknown	1	2.6			
*Years lived in the US*					
Entire life	30	76.9			
More than 5	7	17.9	20	100	21.1 (14,34)
5 or fewer	2	5.1			
*Age at Immigration*					
Less than 18 years old			5	25	
18 years or older			15	75	
Average age (range)					19.9 (13,29)
*Mother’s education*					
Less than high school	19	48.7	16	80	
High school only	11	28.2	1	5	
Post-High school	9	23.1	3	15	
*At least one parent in Agriculture*					
Mother works in agriculture	15	38.5	15	75	
Working, other			3	15	
Not working			2	10	
*Living situation*					
Both parents	22	56.4			
Mother only	14	35.9			
Other	3	7.7			
Number of children					3.4 (1,5)

The research team identified dichotomous labels of “good kids” and “bad kids” de novo in the youth interview data (appendix 1). All youth identified this dichotomous labeling in at least one of their two interviews (59 out of 80 total interviews). All mothers also noted labeling of children as “good” and “bad” in their interviews. Both youth and mothers identified a variety of people who impose these labels on adolescents in the community, including teachers, school staff members, school administrators, peers, people living outside of Salinas, family members, and other parents. Both youth and mothers identified that labels imposed in school can follow a young person over many years.

Youth and mothers also noted that labels can be based on a variety of sources. In some cases, an adult or peer witnesses a specific behavior which is labeled as “good” or “bad”. In many cases labels are passed on through word of mouth from trusted sources or via rumors from wider social networks. Labels also arise by association. Youth reported being labeled based on their neighborhood as well as the school they attend. One youth described an alternative school as,

“A school for … not dumb kids but like kids that are like not going to meet up to their credits so they’re going to have to have like more help… It’s like high school but it’s, yeah, kind of where the bad kids go.”

(13-year-old, US-born girl-identified youth)

“Bad kids” or kids with negative labels might have parents who are perceived to be gang affiliated, on probation, or previously incarcerated. One mother reported,

“I didn’t know what they were up to…they wore red pants…and they dyed their hair red…and they wore a lot of red clothes…because they identified with the Norteños from around here. And…they lack manners. So initially, when they were very little, they played together and visited here. But now that they’ve grown…even their mom dyes her hair red…and red clothing. So, to avoid problems, they stay in their house and we stay in ours. And [my daughter] doesn’t hang out with them or anything.” (53-year-old mother of 4, living in the US for 34 years).

Given this, it was important for mothers to know the parents of their child’s friend. When discussing another friend of her child, this same mother noted,

“I also know the parents. I’ve been there and have talked to them, and that’s how you get to know people, right? So, I’m certain these are good parents and the girls are good girls.”

In such cases children may be labeled based on their parents’ behavior, largely outside of the youth’s control.

### Defining “good” and “bad” kids

#### Good kids

Youth and mothers reported that “good kids”, or kids who are “on the right path” avoid delinquent behaviors such as truancy, consumption of drugs or alcohol, and physical fights. They also abstain from sex according to youth and mothers. When asked to describe “good character”, one youth noted,

“There’s like people that don’t really care about life, they just like, like kind of like, probably, well, like negative, like, yeah. Well, like there’s some people that are like proactive, like, and that if they’re, they really want to do a certain thing they’re going to do it” (13-year-old girl-identified youth, US born).

Some mothers described that “good kids” spend most of their time at home with their families. Mothers also noted that “good kids” are obedient and respectful. One mother reported,

“He helps wash dishes. Ever since he was little, I’ve encouraged him to do something: to clean up, to put his things away. “Help me do the laundry.” He helps me with the soap when I’m washing. He’s always very nice, and he’s always been that way ever since he was little.” (41-year-old mother of 3, living in the US for 20 years).

Participants also described “good” as the absence of “bad”. A mother said,

“But the thing is that the kid doesn’t give me any kind of trouble. He’s a good kid, and his only problem is school performance.” (48-year-old mother of 5, living in the US for 20 years).

“Good kids” communicate frequently with their parents and prefer to spend time with family rather than out with friends. Youth were particularly likely to identify “good kids” as those who participate in sports and in a college prep program in high school. When describing his friends, one youth participant said,

“Right now everything’s going good, I don’t see no signs of their future being harmed…Like I don’t see them starting to think drugs are funny or stuff like that.” (13-year-old boy-identified youth, US born).

Mothers and youth shared similar visions of the trajectories of “good kids.” Mothers focused on the fact that kids “on the right path” would have more choice in their employment in the long run, avoiding the manual labor in the fields that many of these mothers experienced. They also envisioned that “good kids” would have financial stability and may have more opportunities for education. Youth were more likely to specifically mention that “kids on the right path” were college bound and more likely to have more opportunities as well as a “good job” such as one in which they worked indoors. One youth participant said,

“If you’re good you have a lot more like… I don’t know how to say it, you have a lot more opportunities to do things, but when you’re bad, you know, not doing good you really have like less opportunities to do things, you know, less opportunity to go to college, less opportunity to do school activities, and events, and stuff like that.” (13-year-old, girl-identified youth, US born).

One mother reported,

“If you are a good student and you want to get ahead…forget about the money. Don’t worry about that…there’s a lot of help.” And she tells me that as soon as she graduates, she wants to go to college. So I tell her that if she has goals, she needs to let go of whatever might hold her back…she just needs to always move forward.” (53-year-old mother of 2, living in the US for 24 years).

#### Bad kids

Participants described that “Bad kids” or kids on the wrong path are sexually active and engage in delinquent behavior such as skipping classes, drug and alcohol use, and physical fights. Mothers add that “bad kids” spend a lot of time outside of the home. One mother explained,

“A bad friend is… going [out] with them and… my kid doesn’t go out. His friends are the same way.” (48-year-old mother of 5, living in the US for 20 years).

One youth described the consequences of a “bad attitude,”

“They might be involved with drugs or something like that…. like they’re gangsters or something, people that live in the streets…. If they don’t choose to be good they won’t succeed…. To be something or be someone powerful like a teacher or something**”** (13-year-old, girl-identified youth, US born).

Mothers and youth both noted that youth are labeled as “bad” through affiliation with friends or family who are in gangs or engage in delinquent behavior. One youth explained,

“If you’re not a gang member but you hang out with them, you’re going to look like one, guilty like by association.” (13-year-old girl-identified youth, US born).

A mother recalled,

“What affected my kids most was when one of their uncles said, “I don’t want to see you with my kids, because your brother was killed for being a gang member…. I don’t want to see you with my kids.” That’s what they said to my son who’s an engineer.” (46-year-old mother of 4, living in the US for 25 years).

Mothers ascribed “bad kid” labels to friends they did not approve of. In some cases, this manifests as encouraging you to socially isolate from perceived “bad kids”. One mother noted,

“Well, as I said, the [friends that] I know haven’t been negative for her. The ones I don’t know were negative. There was one girl I did meet who I didn’t like at all; I really didn’t want my daughter near her. Because they took her out a lot without my permission. And I told her that I didn’t want that girl near her.” (36-year-old mother of 3, living in the US for 18 years).

Both youth and mothers described the tragedy of making one wrong choice, fearing severe outcomes such as ending up homeless, shot, dead, or in jail. One mother noted,

“Because there are two paths you can choose, and if you choose drugs, you’ll be on the wrong path…you’ll end up in jail…or you’ll get killed…and I don’t want that. You can be better than that.” (37-year-old mother of 4, living in the for 18 years).

Likewise, one youth said,

“Why do you want to be a gangster? You’re just dying for a color, and you shouldn’t – just – just do good in school and then you could be even more rich, and you could travel a lot. And you could have a big house and everything. And the other people, they end up being homeless or dead in the streets or getting shot.” (13-year-old, boy-identified youth, US born).

To protect their children from “being on the wrong track,” mothers adopt a no-margin-for-error philosophy. One mother recalled giving the following advice to her child,

“You have to picture yourself like you’re going up a ladder…and this ladder doesn’t have any railings for you to hang on to. So, if you lose your balance…you’ll fall!” (36-year-old mother of 3, living in the US for 18 years).

Another mother articulated the fear and motivation underlying this idea, stating,

“If we don’t do it that way, then our kids are going to become gang members, get into drugs…Because they’re going to feel like they’re not important to us or like they don’t matter to us… And if we don’t help them, no one else is going to come along with good intentions and tell them, ‘What you’re doing is wrong.’ Quite the opposite: they’re going to be approached by bad people who say, ‘They don’t love you. Let’s go hang out on the street.’” (33-year-old mother of 4, living in the US for 19 years).

### Impacts of labeling

#### Exclusion from peers

Youth talked about how peers who were labeled as “bad” could suddenly be ostracized from their friend groups. One youth said,

“I used to talk to them…they were like good girls, like they just started talking to new people and like they got influenced to skip class and all that. I’m like, ‘Bye,’ just like keep them out, like I don’t want to do that.” (13-year-old girl-identified youth, US born).

Additionally, when discussing advice she gives her children, one mother stated the following,

“Get away from friends like that…kids who are with drugs and weapons…because even if you’re clean and you don’t have any of those things yourselves…just by hanging out with these kids you’re already getting into trouble…because you’re associated with them.” (37-year-old mother of 4, living in the US for 18 years).

#### Limits to educational opportunities

Negative labeling of youth was also found to limit educational opportunities. One youth participant explained the tenacious nature of a “bad kid” label meant that she felt persistently in danger of losing her place in her school.

“I was doing bad at [my middle school] so…they kicked me out of the school, and they put me over there and it’s like independent study…. I used to be really bad, like getting into fights and stuff, so that all stays like on your school record. So, if anything happens, even if I confronted someone, they’re like, ‘Mm, she’s notorious for doing that, like we got to get her out of here.’” (13-year-old girl-identified youth, US born).

One mother described a vicious spiral of her son being impacted by poverty, labeled a “school failure”, and being denied participation in sports:

“They demand a lot from the kids, such as asking them to focus on school. But being poor, we can’t do that…My kid wanted to join basketball, but he couldn’t because he’s not doing well in school…But I really wish they gave him the opportunity to play basketball…Spending more time in school would make him care more about it, that would motivate him to do better. But instead, they’re just penalizing him for not doing well in school, and that makes everything worse. That lowers his self-esteem and makes him care less about school.” (48-year-old mother of 5, living in the US for 20 years).

#### Changes to health-modifying behaviors

Youth participants explained that their health protective-behaviors were compromised at times to preserve their “good kid” labels. For example, youth participants reported that teens in the community were hesitant to use birth control or buy condoms for this reason. One youth said,

“Probably because they feel like, that they would get judged like if they like go to the store by themselves to get like condoms, then the people would be like, ‘Oh, you’re too young,’ and, or like they’ll feel judged. Or like if a girl goes to the doctor and gets like birth control they’ll be like, ‘Oh, you shouldn’t be doing that,” and stuff.” (13-year-old girl identified youth, US born).

Another participant saw the avoidance of sexual health conversations among his peers as an indicator of friend group of “good kids”. When asked why the sexual conversations do not come up within his friend group he said,

“I don’t know, I guess we’re just all good kids, we just focus on sports.” (13-year-old boy-identified youth, US born).

Youth participants also associated certain high-risk health behaviors with “bad kids.” When asked to describe the life of a “bad kid” at the age of twenty, one youth states,

“I’m guessing not having enough money, the girls getting pregnant, broke, being a drug addict. Or being killed or sent to jail.” (13-year-old boy-identified youth, US born).

Another youth who had been labeled and self-identified as a “bad kid” explained how this led to cycles of involvement with law enforcement and engagement with drugs,

“And, yeah, and then, I don’t know, I just started running away, kept on selling drugs, doing all that. The experiences that I have with the law is horrible. I’ve been in handcuffs more than twice, more, Jesus, yeah, I was a mess.” (13-year-old girl-identified youth, US born).

### Resistance to labeling: close relationships

Labeling was challenged when the subject of the labeling was research participant or someone in close social proximity to them such as their friend or child. For example, mothers were more likely to describe a “good” kid who did a “bad” thing when they were talking about their own child. When talking about her son who recently started to exhibit “bad kid” behavioral change such as skipping class and becoming “more rebellious” and “little lazier”, one mother mentioned,

“He’s an only child. But it’s gotten really complicated for me. To see all the changes in him, and to see how he’s doing step by step; it seems like he’s going down instead of up.” (32-year-old mother of 1, living in the US for 18 years).

Mothers and youth described instances of wanting to challenge a label given to a young person in close social proximity to them. Some youth challenged negative labels within the school setting. One youth challenged the negative labels from school employees applied to her friends though she seems to have internalized the bad label applied to her:

“Some staff, like, because I’ve been getting in trouble, they say that [my friends are] probably not good people… because people just see the bad of things on them but if they get to know them, like they’re, they’re amazing people.” (14-year-old girl-identified youth, US born).

### Resistance to labeling: community reputation

While most instances of labeling were applied at the individual level we did identify instances of labeling at the community level. More than half of the youth challenged negative labels imposed on their city’s reputation through local and regional news sources or word of mouth, making a point to underscore the positive aspects of their community. Regarding Salinas, one youth said,

It’s a nice place to live but sometimes it’s a little dangerous. It’s a nice place to be at because it has many places you can go to. And it’s a really nice place, sometimes in the mornings, you can see the mountains and it’s really beautiful to just look at it… there’s some bad stuff but there’s too some good stuff it has.” (14-year-old girl-identified youth, born outside the US).

Youth discussed fallacies that can arise with negative labeling, alluding to injustices of reducing Salinas to single narrative, often imposed by an outsider viewpoint. One youth noted,

“I’ve never felt like bad being in a neighborhood, like it can be, like I said, [one neighborhood] where everyone says like, ‘Oh, it’s dangerous,’ but I’ve never really felt like I’m in danger or anything….I think it’s just because I grew up here and I feel like everyone outside of Salinas just like makes it like, just adds like more like than what it actually is.” (13-year-old boy-identified youth, US born).

When asked about the future, some youth aspire to use their future successes as attestation that their community is more than its negative reputation. One youth noted,

“ I think like people my age, referring to my friends, I think we all see like each other as like someone big, like we want to impact the community and graduate from college, and most of us want to come back here to help the community… [we] want to come back to the community and do all these great things and showing people that Salinas is a great community.” (14-year-old girl-identified youth, US born).

## Discussion

This study draws from in-depth interviews with early adolescents and mothers in a in Salinas, California to demonstrate the pervasiveness of formal and informal “good” and “bad” labels placed on Latinx adolescents in one Californian agricultural community, and variety of ways that these labels influence youth engagement in education and health protective behaviors. Dichotomous labeling was an emergent theme that arose organically in interviews with all the adolescent and mother participants, reflecting the lived experiences of our participants in their community. To this end, taking an inductively qualitative research approach, treating our participants as experts in their community and lived experiences, transforms the research relationship as it challenges hierarchical systems of knowledge, and positions participant as a co-producer of knowledge. Our participants named both the short-term and long-term consequences of the exclusionary labels they witnessed and experienced. In the short-term participants described denial from sports, academic disengagement, school removal, and health-modifying behaviors in the form of drug use and hesitancy in birth control and condom use. Long-term consequences were described as undesired pregnancy, incarceration, gang involvement, and being shot or killed. This adds to the very limited research that examines the public health implications associated with being labeled or avoiding a label.

### Negative labels fostering exclusion

Participants in this study described the negative labeling of youth as an exclusionary practice that jeopardizes supportive relationships among peers and adults, community connectedness, and school engagement. Specifically, our participants discussed exclusion from social groups and formal school discipline. These findings are in alignment with prior studies on the impact of school disciplinary practices and policies, which disproportionately target minoritized youth [29, 30]. In such cases, school labels, operationalized through expulsion, suspension, or disciplinary action, follow students’ long term. Studies have shown that being pushed out of school results in a cascade of negative outcomes that ultimately increases the risk for involvement in the criminal justice system [31–33]. Short of criminal justice involvement these exclusionary practices can reduce school connectedness. which is protective for a variety of health and mental health outcomes [34]. Our own research in this population has shown that low school connectedness is associated with higher depressive symptoms [4]. More research is needed to better understand the disparate association between punitive and exclusionary labels and low wealth, socio-politically oppressed communities to create source-driven and sustainable solutions.

### Labeling: responding to social exclusion

Both youth and mothers in this study emphasized the importance of “staying on the right path” and the literal loss of life and future opportunity for young people who receive negative stigmatizing and exclusionary labels. As noted, one mother described a ‘ladder without railings’ articulating a sentiment of there being no room for mistakes, a prevailing message echoed from several youth and mothers in our study. One could hypothesize that strict conception of “good” and “bad” is one form of no-margin-for-error messaging given conditions of other exclusionary policies such as over-policing and criminalization of Latinx immigrants communities as previously described in the literature [35, 36]. From a liberation psychology lens, conditions, such as systemic and structural forces that promote sociopolitical exclusion, poverty, and resource scarcity, give rise to psychological patterns that often reinforce conditions of internalized oppression [37, 38]. Literature on “legal consciousness,” a perspective infused most fundamentally with stigma and fear, describes internalization of immigration status among first-generation undocumented immigrants [39, 40]. Immigration status as a form of labeling powerfully informs how people see themselves and their rights in the United States, internalizing the notion that they have no rights. Like the role of immigration status seen in legal consciousness, fear and stigma predominates in “bad” labeling due to its associated social exclusion. To this end, it is possible to address internalized labels harbored by marginalized communities and mobilize a path to liberation by targeting and mitigating causes of stigma and fear and dismantling the conditions that uphold these conditions.

### Resisting labels and promoting social belonging

Interestingly, many participants in this study voiced nuances in dichotomous labeling. First, we saw many participants resist negative labeling when it was imposed on those close to them, such as friends or their own children. Second, we saw a similar phenomenon when some participants in our study challenged negative labels imposed on Salinas, making it point to contrast these labels with positive attributes of their community. Reframing the outsider, deficit-focused narrative regarding Salinas also arose throughout our study design and implementation with our community-based partners. Although these two examples of participants challenging negative labels placed on people and community that are close to them may appear as simple social phenomenon, they also present a psychological lever to combat negative labeling and underscores the potential in promoting social belonging. It is well studied that social rejection, often disproportionately felt by marginalized youth, have both mental and physical consequences [19, 20, 41, 42]. Social-psychological interventions aim at improving sense of social belong have been shown to improve academic outcomes and mental well-being [43–46].Our participants who are resisting negative labeling and attempting to preserve belonging for their community and those they view as socially close, add to the compelling body of evidence that recognizes the importance of fostering a sense of belonging and strengthening connection among school personnel, students, families, and community members. An approach focused on belonging may also provide a path for schools and youth-serving institutions to avoid inadvertently conflating asset driven or strength-based approaches with “good kid” labels given that anywhere children are labeled as “good” implies the existence of dichotomous “bad kids.”

### Implications for framing public health research questions

The adolescents and mothers in this study highlight the social and health consequences of negative labels placed on Salinas youth and the stigma associated with negative labels placed on the Salinas community. Similarly, *A Crecer’s* community advisory board, youth advisors, and community co-investigators have all continuously challenge the A Crecer study team to investigate ways that community assets might be leveraged to address the community’s challenges rather than just reiterate epidemiologic “risks.” Together these approaches are related to the literature on the insidious impact of deficit-based approaches in public health and research which has given rise to a movement away from damage-centered research where depletion, brokenness, and hopelessness are used to leverage concern, resources, and reparations for marginalized communities [47–49]. Common labels within public health research such as “resource-scarce”, “poverty-stricken” and “at-risk” can pathologize communities and further erode trust and relationship-building with public health and academic institutions [50, 51]. A potential strategy to combat this deficit-centered labeling of communities like Salinas is asset-framing— which emphasizes the strengths, aspirations and dreams over challenges and the benefits of investing in these communities for positive change [52, 53]. This approached does not ignore the presence of public health challenges but instead attempts to reduce the harm of stigmatizing labels and foster additional opportunities for exploring avenues of intervention and change. More research rooted in asset-based framing is needed not only to better to assess its potential as an advocacy tool, but also equitably target structural and systemic causes of disparities in health and social outcomes.

### Limitations

This study has several limitations. While the framing of “good kids” and “bad kids” arose from our participants, it is inherently dichotomous and reductionist, failing to capture the complexity of the human condition including the environmental factors which drive human behavior and the ways that adolescents change and grow overtime. Our sample reflects the experiences of a single community, limiting its broad generalizability though these findings are likely transferable to related contexts. All our participants self-identify as Latinx, thus our understanding of how labeling operates between racial and ethnic groups is limited. The denovo emergence of labeling as a theme meant more targeted and in-depth questions were not asked. To this end, moral and cultural dimensions prevalent among Latinx communities such as machismo, marianismo, religion, and gender roles and their relationship to labeling could not be assessed. Additionally, we were not able to fully explore the importance of factors such as socioeconomic status, parental education, immigrant documentation status, and skin color. Nonetheless, this study adds to the limited literature on labeling and is urgently needed to better understand these understudied youth.

## Conclusions

This study extends Labeling Theory to understand the public health implications of labeling adolescents within a primarily Latinx agricultural community. Formal and informal “good” and “bad” labeling of youth had implications for youth engagement and health-protective behaviors and educational pursuits. Social connection allowed youth and parents to challenge negative labels, which offers compelling evidence against exclusionary practices and in favor of practices that foster a sense of belonging and strengthen connections among school personnel, students, families, and community members. Our participants also highlighted the harm in damaged-centered community narratives prescribed to communities like Salinas, calling us to consider how to incorporate more asset-framing and strengths-based frameworks in public health work. This study’s findings can help public health practitioners better understand the perceptions and experiences of youth from immigrant and Latinx communities, which can aid in optimizing public health interventions that seek to improve the health, well-being, and academic success of youth.

## Supplementary Information

Supplementary Material 1.

## Data Availability

The datasets generated and/or analyzed during the current study are not publicly available to protect the privacy of study participants but are available from the corresponding author upon reasonable request. Competing Interests: The authors have no conflicts of interest or financial interests relevant to this article to disclose.

## Abbreviations

ACEs: Adverse Childhood Experiences
US: United States
CA: California
RTI: Research Triangle Institute
UCSF: University of California San Francisco
