# Supplementary material for: Labeling in a California Latinx community: public health implications for youth and role in community narratives
Source: BMC Public Health. 2025 Jul 23;25:2536. doi: 10.1186/s12889-025-23598-z (PMC12285011; doi:10.1186/s12889-025-23598-z)
Supplement: Supplementary file 1 — Supplementary Material 1. [file 12889_2025_23598_MOESM1_ESM.docx]

Appendix 1.

| Code Table: Labeling Code Categories, Definitions, and Examples From Youth and Latinx mothers | | | | |
| --- | --- | --- | --- | --- |
| **Code** | **Definition** | **Subtheme** | **Description Summary** | **Example** |
| Sources of labels | Origin of a label prescribed to an individual as a means to classify or categorize them into a particular group | Observed behaviors | Categorization of individual based on observation of their behavior | P: She’s good with her time: when’s time to do homework, she does it; when’s time to go play in the back yard, she goes; when she has to take her dog out, she does that; then she showers...and there...she has her routine.” (53-year-old mother of 4, living in the US for 34 years) |
|  |  | Rumors | Categorization of individual based on a circulating story | “P: Maybe if I heard she had a friend who had a problem, I wouldn't want her to have that friendship. I would try to gradually separate her from that friend. But I haven’t heard anything bad about the friends she has.” (53-year-old mother of 4, living in the US for 34 years) |
|  |  | By association | Categorization of an individual based on association with such as a peer, a social group, community, family members, etc | “A lot of kids that are my age are obviously into like gangs and stuff, so what I mean by like being careful is you don’t want to hang out with those kind of people because you don’t have to be a gang member to be like affiliated or to look like one..” (13-year-old girl-identified youth, US born) |
| Positive Labels | Behaviors, character traits, trajectories or conceptions of a person that are consider positive or good | Behavior | Positive behaviors were described as the youth who are not sexually active, do not engage in fights, school-focused, involved in extracurricular activity, helps the household and stays with their family. | “He’s very responsible. Since he was a little child, I’ve asked him to help me around the house. So, he helps me with lots of chores, such as sweeping, dusting, washing dishes, all that. And the truth is that he’s always the more responsible one. Whenever I ask him to help me with a chore, he just does it.” (37-year-old mother of 4, living in the US for 18 years) |
|  |  | Characteristics | Positive labels were described as motivated, hardworking, giving, respectful, quiet, and obedient | “But [she] is very reserved” She’s not the type to go out dancing on the weekends. No...her friends are...very reserved and focused in school. She knows how to choose her friends. When she comes home...she just eats...then she does her homework...then she asks for my permission to watch TV...she asks for my permission for just about everything. And I let her...and she’s a very good daughter.” (53 year-old mother of 2, living in the US for 24 years) |
|  |  | Affiliations | Positive affiliation was described as having “good” friends where you can meet their parents, being on a sport’s team or school clubs, and having close relationships with family | “When a friend says, “I want to study. I don’t want a boyfriend because I want to stay focused and be someone in life. Because my sister went to college and I want to go, too.” And she tells them, too, “My brother got his degree. I want to get mine, too, and be someone in life. Let’s study hard, and let’s see what programs there are and everything.” And they encourage each other.” (46-year-old mother of 4, living in US for 25 years) |
|  |  | Future projection | Projections for kids with a positive label were college-bound or higher education, getting good jobs that do not require manual labor, having a number of work options or available opportunities, and achieving financial stability | “If you are a good student and you want to get ahead...forget about the money. Don’t worry about that...there’s a lot of help.” And she tells me that as soon as she graduates, she wants to go to college. So I tell her that if she has goals, she needs to let go of whatever might hold her back...she just needs to always move forward.” (53-year-old mother of 2, living in the US for 24 years) |
| Negative Labels | Behaviors, character traits, trajectories or conceptions of a person that are consider negative or bad | Behavior | Negative behaviors included engaging in sexual activity, pregnancy, drug use, skipping classes, fighting, laziness, not helping around the house, having a lot of friends, and going to parties or staying out late | “It’s much worse now that marijuana is legal. It’s gotten worse. You see teenagers, boys and girls, who are high and everything. And as parents, it’s hard to see… what’s it called… to see teenagers hanging out on the street like that. As a mother, I wouldn’t want to see my kids like that.” (46-year-old mother of 4, living in the US for 25 years) |
|  |  | Affiliation | Negative affiliation included being in a gang or having family members or friends in a gang, parents who work too much, having “bad kid” friends or kids that go to alternative schools | P: Well, I think when they hang out with certain kids who have a different mentality, like they don’t want to study; they want to find something else to do, like smoking and things like that. I think they’re the ones who ruin the other ones who are doing well. Especially the boys if they pay attention to them. Because, as I tell my son, “If you pay attention to them, you’re going to end up like them.” –(41-year-old mother of 3, living in the US for 20 years) |
|  |  | Future projection | Projections for kids with negative label included the lost of opportunities and resources as well as convergence onto a path that could result in death, jail, or shot, with limited chances to change. | “If you are a good student and you want to get ahead...forget about the money. Don’t worry about that...there’s a lot of help.” And she tells me that as soon as she graduates, she wants to go to college. So I tell her that if she has goals, she needs to let go of whatever might hold her back...she just needs to always move forward.” (53-year-old mother of 2, living in the US for 24 years) |
|  |  | Proximity Impact | Closer an individual is to another person or a community, the more nuance their perception of a label or more willing to challenge a label, this included parents noting a “good” kid could do “bad” when talking about their own children, a youth participant describing a friend who has a misstep but can still aspire to go to college, or a participant challenging negative perceptions of their community. | “They sometimes follow bad tracks, like behave bad in school, but they still get good grades. They also want, go to college and university.” (13-year-old boy-identified youth, born outside the US) |
| Impact of Labels | Outcomes associated with a given label | Reduction of health protective behavior | To avoid being a negative label, youth participants described birth control use hesitancy, reduction in condom use, avoidance of sexual and reproductive health conversations. | “ I think, well, most of the girls here, like if they do have a friends with benefits then they’re either called like a bad name, I guess for the guy and the girl, you know, like they call them like names and they just think bad of you, or they think bad of a person” (14 year-old girl-identified youth, born outside the US) |
|  |  | Disengagement from parental or community support | Kids labeled as “bad” are remarked and discouraged by school officials, parents and community members | “Then another mom told me: “I’m worried about [child’s name], because that girl...my daughter used to spend time with her...and people say she likes to use drugs.” The woman told me that, so then I said I wouldn’t let her...hang out with that girl. Even though she seems like a good girl, but I’m not letting her” (53-year-old mother of 4, 34 years in the US) |
|  |  | Ostracism from peer support | Youth or parents leverage labeling of other youth to distance themselves from “bad” kids. | “Get away from friends like that...kids who are with drugs and weapons...because even if you’re clean and you don’t have any of those things yourselves...just by hanging out with these kids you’re already getting into trouble...because you’re associated with them.” (37-year-old mother of 4, living in the US for 18 years) |
|  |  | Limited education opportunities | “Bad” kids have fewer educational opportunities and limited access to school activities | “Your citizenship is something like you give your teacher, like they grade you on that, like respectful or whatever, I don’t even know what that means actually. And like ‘O’, outstanding, ‘S’, satisfactory, and ‘U’ like bad. And there’s like this thing in middle school, like if you get more than one ‘U’ you can’t promote. “  (14-year-old girl identified youth, US born) |
